# Does family planning counseling reduce unmet need for modern contraception among postpartum women: Evidence from a stepped-wedge cluster randomized trial in Nepal

**DOI:** 10.1371/journal.pone.0249106

**Published:** 2021-03-26

**Authors:** Mahesh Chandra Puri, Sarah Huber-Krum, David Canning, Muqi Guo, Iqbal H. Shah

**Affiliations:** 1 Center for Research on Environment Health and Population Activities (CREHPA), Kathmandu, Nepal; 2 Harvard T.H. Chan School of Public Health, Boston, Massachusetts, United States of America; University of Alberta, CANADA

## Abstract

**Background:**

Postpartum women have high rates of unmet need for modern contraception in the two years following birth in Nepal. We assessed whether providing contraceptive counseling during pregnancy and/or prior to discharge from the hospital for birth or after discharge from the hospital for birth was associated with reduced postpartum unmet need in Nepal.

**Methods:**

We used data from a larger a stepped-wedge, cluster randomized trial, including contraceptive counselling in six tertiary hospitals. Group 1 hospitals (three hospitals) initiated the intervention after three months of baseline data collection, while Group 2 hospitals (three hospitals) initiated the same intervention after nine months. We have enrolled 21,280 women in the baseline interviews and conducted two follow-up interviews with them, one and two years after they had delivered in one of our study hospitals. We estimated the effect of counseling and its timing (pre-discharge, post- discharge, both, or neither) on unmet need for modern contraception in the postpartum period, using random-effects logistic regressions.

**Results:**

Unmet need for modern contraception was high (54% at one year and 50% at two years). Women counseled in either the pre-discharge period (Odds ratio [OR] 0·86; 95% CI: 0·80, 0·93) or in the post-discharge period (OR 0·86; 95% CI: 0·79, 0·93) were less likely to have an unmet need in the postpartum period compared to women with no counseling. However, women who received counseling in both the pre- and post-discharge period were 27% less likely than women who had not received counseling to have unmet need (OR 0.73; 95% CI: 0·67, 0·80).

**Conclusions:**

Counseling women either before or after discharge reduces unmet need for postpartum contraception but counseling in both periods is most effective.

## Introduction

In developing countries, 218 million women want to space or limit their pregnancies but are not using a modern method of contraception [1]. Women with an unmet need for modern contraception account for 77% of all unintended pregnancies [1]. Generally, women are considered to have an unmet need for family planning (FP) if they are sexually active and want to avoid pregnancy but are not using any method of family planning (while unmet need for modern contraception refers to use of modern methods only). By helping women to prevent unintended pregnancies, programs can reduce unintended births and unsafe abortions, and improve maternal and child health [2, 3]. These gains can also contribute to other development objectives, such as curbing poverty and slowing population growth [4, 5]. Therefore, improving access to contraceptive methods and reducing unmet need are primary objectives of FP programs and policies and key to achieving global public health goals, such as the Sustainable Development Goals (SDGs) on sexual and reproductive health [6].

The World Health Organization (WHO) recommends waiting at least 24 months after a live birth and six months after a miscarriage or induced abortion to conceive another pregnancy in order to reduce the risk of adverse maternal, perinatal and infant outcomes [7]. FP can potentially avert over 30% of maternal deaths and 10% of child mortality [8, 9]. Despite the steep rise in contraceptive use in several developing countries, inter-birth intervals have increased only slightly over the past 25 years: 25% of second and higher-order children are born within two years of a sibling, compared to about 29% a decade earlier [10]. Birth intervals are often short primarily because of a poor knowledge of health risks associated with short pregnancy intervals and low uptake of contraceptives during this period [8, 9].

Reasons for overlooking the use of contraception during the postpartum period may be due to inadequate counseling about FP during the antenatal and/or postpartum period [11]. Integration of FP counseling into maternal and postnatal services is a cost-effective way to inform women about the risks of short-birth intervals and the benefits of contraceptive use [12]. However, while counseling in the postpartum period is positively associated with postpartum contraceptive use [13, 14], empirical evidence is inconclusive on whether FP counseling during antenatal care is associated with contraceptive use in the postpartum period [15–17]. Further, past studies tend to focus on contraceptive uptake, mostly in the year post-delivery, rather than reduction of unmet need for postpartum FP [13–17].

In Nepal, FP programming continues to be a priority of the Ministry of Health and Population as evident by the country’s commitments to the FP 2020 program, the family planning targets of the SDGs, and the preparation of National Family Planning Costed Implementation Plan 2015–2020 [18]. Use of modern FP among married women aged 15–49 years increased from 26% to 43% between 1996 and 2016, and unmet need for FP decreased from 34% to 24% over the same period [19]. In addition, the median duration of postpartum amenorrhea fell from 11·1 months in 2001 to 6·0 months in 2016, while the median duration of postpartum insusceptibility declined from 11·4 to 7·8 months [19].

Over 50% of women in Nepal have an unmet need for postpartum FP [19, 20]. In Nepal, 25% of women become pregnant within 24 months postpartum [19]. This suggests there are missed opportunities to provide contraception counseling to postpartum women. Given the increasing trend of antenatal care visits (84% in 2016) and health facility deliveries (57% of all births in 2016) in Nepal, integration of FP counseling at the time of antenatal care and delivery has the potential to increase postpartum contraceptive use and reduce unmet need. However, FP counseling is traditionally provided during postnatal care in Nepal, but implemented infrequently: approximately 13% of women are provided information about FP during postnatal care [19].

This paper examines whether providing family planning counseling during (a) antenatal health care visits, (b) postnatal health care visits, or (c) both is associated with unmet need for modern contraception during two years postpartum among women in Nepal. We used secondary data from a larger study that aimed at evaluating the effects of an intervention aiming to institutionalize FP counseling during routine maternity care services. Further, we were interested in understanding the socio-demographic correlates of unmet need for modern contraception during the postpartum period.

To meet the objectives of the paper, we utilized panel data of women enrolled at the time of delivery and followed up at one and two years later. We measure the impact of counseling on unmet need for modern contraception over two years postpartum period. Most other studies focus on contraception during one year postpartum. Variables included in our study are based on a review of literature and, in addition, take into account the socio-cultural context of Nepal. The paper, therefore, aims to better capture the evolving level of unmet need over an extended postpartum period of two years for a cohort of women who delivered in six study hospitals and provides insights not covered in other studies.

## Materials and methods

### Study design and clusters

In this stepped-wedge, cluster-randomized trial, we assessed an externally supported, family planning program [21]. The program, the Postpartum Intrauterine Device (PPIUD) Initiative, was implemented by the International Federation of Gynecology and Obstetrics (FIGO) in collaboration with its national societies. The PPIUD Initiative was launched in six countries, including Nepal, in 2013. The aim of the Initiative was to institutionalize contraceptive counseling during routine antenatal care services and at delivery and the availability of PPIUD as a routine part of maternal health services [21]. In Nepal, FIGO collaborated with the Nepal Society of Obstetricians and Gynecologists (NESOG) and the Nepal Ministry of Health and Population to design the intervention in adherence with the national health system and training guidelines. The intervention involved five components:

1. female community health volunteers and hospital staff were trained on postpartum contraception,
2. maternity care providers were trained on counseling and PPIUD insertion and complications management,
3. counseling aids and informational tools, specially leaflets, wall charts, and videos, were provided and distributed during counseling and displayed in waiting areas,
4. Kelly’s forceps for vaginal PPIUD insertion and PPIUDs were provided, and
5. one provider in each hospital was designated as the facility coordinator for the program to provide on-going support for the initiative.

Providers were trained to counsel women during routine antenatal care, at early labor, and after delivery but before discharge from the hospital. Providers received refresher trainings, as needed, and were expected to train rotated-in providers. Counselors were expected to provide information about all available FP methods to women and to demonstrate how the PPIUD is inserted using visual aids. Pregnant women who received counseling in the antenatal period had the option to provide advance consent to PPIUD insertion, and their medical charts were marked with their stated decision. These women were consented again at the time of delivery to confirm their choice for PPIUD insertion.

Contraceptive counseling and commodities, including male condoms, pills, injectables, IUDs, implants, and sterilization, are provided by the Government of Nepal at no cost. IUDs were already included in Nepal’s method mix; however, the method is only available at a limited number of hospitals, where trained providers are available. Thus, PPIUD services, including the device, insertion and removal, were offered to women free of charge. Removal information was covered in the counseling, and providers instructed women to return to the facility at any time if they wanted the PPIUD removed.

The evaluation of the FIGO PPIUD intervention in Nepal was conducted in six hospitals: Bharatpur Hospital, Bheri Zonal Hospital, BP Koirala Institute of Health Sciences (BPKIHS), Koshi Zonal Hospital, Lumbini Zonal Hospital and Western Regional Hospital. The inclusion criteria for hospital sites were: (1) high volume of obstetric caseloads (>6,000 per year), (2) large catchment area, and (3) not located inside of the capital city of Kathmandu. Tertiary hospitals, rather than smaller, primary health centers, were of focus because of feasibility.

Details about the study design have been published by Canning et al. (2016) and the trial has been registered with ClinicalTrials.gov, NCT02718222 [22]. Ethical approval as exempt was granted by the Harvard T.H. Chan School of Public Health Office of Human Research Administration (Protocol IRB 15–1035), as only de-identified data were provided to Harvard for analysis. The study received approval from the Nepal Health Research Council (Reg No 51/2015).

### Randomization and masking

The evaluation followed a stepped-wedge cluster randomized design, consisting of a baseline survey of women after delivery but before discharge from the hospital and two follow-up surveys, approximately one year and two years after delivery. The six hospitals were pair-wise matched based on geographic location and annual delivery caseload following which one hospital within each pair was randomly assigned to Group 1 and the other to Group 2. The three pairs were: (i) Western Regional and BPKIHS, (ii) Lumbini Zonal and Bharatpur, and (iii) Koshi Zonal and Bheri Zonal. Group 1 hospitals were Lumbini Zonal, Koshi Zonal, and Western Regional. Baseline data collection started in all six hospitals at the same time, September 8, 2015, and ended on the same date, March 8, 2017. Group 1 hospitals initiated the intervention after three months of baseline data collection, while Group 2 hospitals initiated the same intervention after nine months of baseline data collection.

### Procedures

All women who gave birth in study hospitals in the 18-month study period and whose primary residence was in Nepal were eligible for participation in the baseline survey. After giving birth and before discharge from the hospital, site-based trained Nepali female enumerators approached women, introduced themselves, informed women they were conducting a study about postpartum family planning, and screened them for eligibility. Women were provided details about the study in their own language, including the nature of the study, research objectives, benefits and risks, contact information for study investigators, and how their privacy would be maintained. The informed consent script was read aloud to women. Enumerators asked participants to provide written consent to take part in the study. Participants who were unable to sign their names provided a thumbprint. Women were not provided compensation for baseline interviews.

Enumerators interviewed women in their spoken language using hand-held tablets. Interviews were conducted in private locations; only the enumerator and the respondent were present during the interview. Enumerators were instructed to end the interview or change the discussion if anyone interrupted the interview and/or entered the private location. During the introduction module of the survey, participants were told that they could skip any question they did not want to answer or withdraw from the interview any time if they wanted to. The baseline survey included questions about women’s socio-demographic background, reproductive and contraceptive history, antenatal contraceptive counseling, and uptake of PPIUD. At the end of the survey, women were given the opportunity to make comments and ask questions.

The year one follow-up survey occurred between May 30, 2016 and April 30, 2018. The year two follow-up survey occurred between March 17, 2017 and December 31, 2018. The baseline study was powered to detect an effect size in the primary outcome (i.e., PPIUD uptake) of five percentage points [22]. We assumed that 20% of women would discontinue the PPIUD during the intervention, and thus powered to detect an effect size in PPIUD continuation of 3·96% in the follow-up surveys [22]. The sampling frame for follow-up among women who did not get a PPIUD inserted was limited to those who lived within 24 hours travel distance by public transportation of the hospital at which they delivered with the aim of reducing travel costs of follow-up. Based on the power calculation and in consideration of the costs of follow-up, 36% of women who met inclusion criteria and did not have the PPIUD inserted at baseline were randomly selected for follow-up. All women who met inclusion criteria and had the PPIUD inserted at baseline were selected for follow-up to measure continuation, expulsion, and side effects. In total, 26,222 women were selected for follow-up (i.e., 100% of women who had a PPIUD inserted and 36% of women who did not have a PPIUD inserted and lived within 24 hours of the hospital at which they delivered). We adjusted for sampling bias in our estimates using sampling weights (described in more detail below); our weighted sample was therefore representative of women living within 24 hours travel distance of the hospital at which they delivered.

Trained Nepali female enumerators contacted women selected for follow-up to schedule interviews. We followed the same consenting and privacy procedures as the baseline, including written consent. Participants who were unable to sign their names provided a thumbprint along with a witness’ signature. Women were provided 340 Nepalese Rupees (equivalent to $3 USD) as compensation for their participation in follow-up surveys. Interviews occurred in private settings in or near women’s homes and in their spoken language, using hand-held tablets. The follow-up surveys included questions about women’s socio-demographic background, reproductive and contraceptive use history, uptake of contraception, and satisfaction with contraception.

### Outcomes

Our key outcome measure was unmet need for modern contraception measured at each follow-up. Modern contraceptive use was defined as male or female condoms, oral contraceptive pills, emergency contraception, injectable Depo-Provera, sub-dermal implants, IUDs, or male or female sterilization at the time of the follow-up survey [23].

Women were categorized as having unmet need for modern contraception if they had an unmet need for limiting childbearing or an unmet need for spacing pregnancy at the time of the follow-up survey. Women had unmet need for limiting childbearing if they were: (1) pregnant and did not want any more children or they reported they were unsure about having more children, (2) postpartum amenorrheic and did not want any more children or they reported they were unsure about having more children, or (3) fecund, not pregnant or postpartum amenorrheic, did not want more children or they reported they were unsure about having more children, and not using a modern method of contraception. Women were categorized as having unmet need for spacing if they were: (1) pregnant and wanted another child later, (2) postpartum amenorrheic and wanted another child later, or (3) fecund, not pregnant or postpartum amenorrheic, wanted another child later, and not using a modern method of contraception. These definitions are consistent with those used by the Demographic and Health Surveys Program [24].

The primary predictor variable for this analysis was exposure to and timing of counseling about modern contraception. We categorized counseling into four distinct groups: no counseling received (none), counseled prior to discharge from the hospital (pre-discharge), counseled after discharge from the hospital (post-discharge), and counseled both prior to and after discharge from the hospital (Pre- and post-discharge). We use the term “pre-discharge” rather than “antenatal”, because women could have been counseled about contraception during their pregnancy and/or after admission to the hospital for delivery but prior to discharge. We use the term “post-discharge” rather than “postnatal”, because women could have been counseled after delivery but in the postnatal ward. This counseling could be considered “postnatal counseling”, but in this analysis we distinguished between counseling by hospital discharge. We made this decision based on how questions were worded (see below).

To determine whether women received pre-discharge counseling, we asked women two questions at baseline, including: “Were you counseled about family planning/birth spacing during your pregnancy?” and “Were you counseled about family planning/birth spacing after admission for delivery to the hospital?” If women self-reported that they had been counseled, they were asked in an open-ended fashion to detail which methods they were counseled about. Women were able to provide more than one response, and interviewers could indicate on the form whether the woman was counseled about female sterilization, male sterilization, IUD, injectables, implants/Norplant, pill, condom, emergency contraception, calendar method, lactational amenorrhea method, withdrawal, or other (and specify other). To determine whether women received post-discharge counseling, we asked women one question at both follow-ups surveys: “Since leaving the hospital after the birth of [name of index child], have you received information or counseling on using postpartum contraception?” If women responded ‘yes’, they were asked to detail which methods they had counseled about and where they received this information. Women were considered to have received post-discharge counseling if (1) they were counseled about a modern contraceptive method and (2) if they received the information in a hospital or health facility. We note that the counseling women received either during pregnancy and/or after admission for delivery to the hospital was conducted by hospital staff participating in the intervention.

### Statistical analysis

We used Stata version 15 to manage and analyze the data (StataCorp LLC, College Station TX, USA). We limited the analysis to only women who lived within 24 hours distance to the hospital at which they delivered. Since the probability of being followed-up varied by PPIUD insertion status, we calculated sampling weights to create a sample which was representative of the study population at large. Sampling weights were defined as the inverse of the probability of being selected for follow-up, if selected for follow-up, based on a model of follow-up on baseline characteristics (S1 and S2 Tables and S1 and S2 Figs). For women who did not get the PPIUD inserted at baseline, their predicted probability of follow-up was multiplied by 0.36 to account for the fact that only 36% of this population was selected for follow-up. We conducted the analysis by fitting weighted random-effects logit models. We fit models both with and without women-level socio-demographic covariates. All regression models included hospital and month fixed effects to control for time-invariant hospital effects and underlying time trends, respectively. Statistical significance was set at *p* < .05, with 95% confidence intervals.

## Results

**Fig 1:**
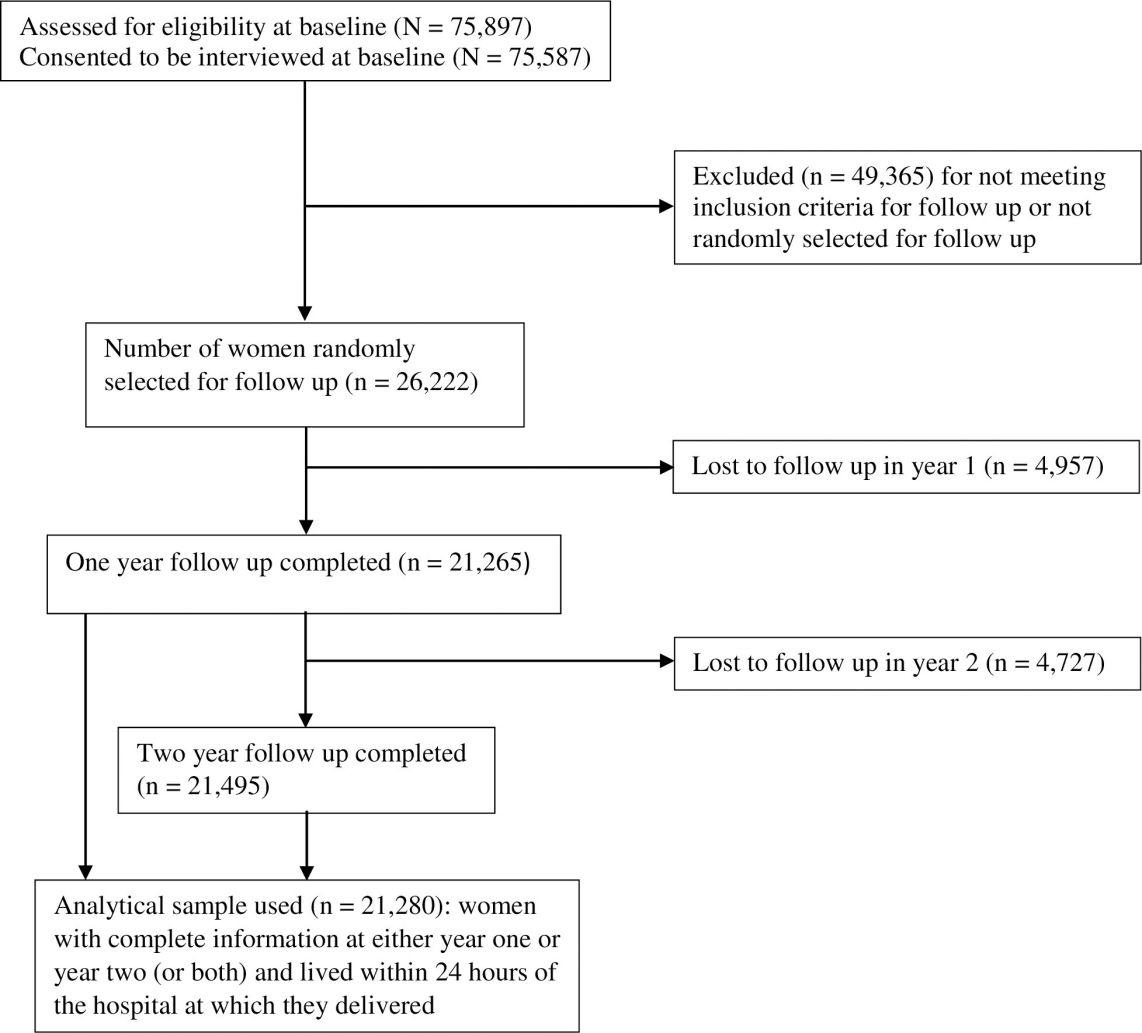
Consort flow diagram.

Of the 75,897 eligible women, 75,587 (99·6%) consented to be interviewed for the baseline survey. Of the 26,222 women selected for follow-up, the response rate (i.e., rate of contact and consent) for year one was 81·1% (n = 21,265) and for year two was 82·0% (n = 21,495). The analytical sample used in this study consists of 21,280 women with complete information at either year one or year two (or both) and lived within 24 hours of the hospital at which they delivered (Fig 1).

Table 1 presents the selected socio-demographic characteristics of women, by the baseline and follow-up surveys. At baseline, a large proportion of women were between the ages of 21 and 25 (43·7%) and over half had only one pregnancy (52·9%). Over half of women at baseline had received secondary or higher level of education (54%). About one-third of women had ever used any method of family planning at baseline. At follow-up, approximately one-fourth of women reported that their husband was away. In the Nepalese context of son preference, we also looked at number of living sons. The majority of women had a son alive at year one and year two follow-up, 65·7% and 66·3%, respectively. Few women reported that the index child had died since giving birth (1·5% at year one and 1·8% at year two).

**Table 1:** Participant background characteristics, number and percentage of baseline, year one, and year two samples.

Characteristic	Baseline (n = 21280)		Year 1 (n = 17804)		Year 2 (n = 17809)	
	N	%	n	%	n	%
**Baseline characteristics**						
**Age at baseline**						
<21	4703	22·1	3913	22·0	3781	21·2
21–25	9294	43·7	7640	42·9	7716	43·3
26–30	5471	25·7	4663	26·2	4719	26·5
31–35	1521	7·2	1335	7·5	1341	7·5
>35	291	1·4	253	1·4	252	1·4
**Parity at baseline**						
1	11252	52·9	8957	50·3	9032	50·7
2	7624	35·8	6704	37·7	6674	37·5
3 or more	2404	11·3	2143	12·0	2103	11·8
**Age at first marriage**						
<20	11118	52·3	9472	53·2	9334	52·4
20–24	8598	40·4	7077	39·8	7185	40·3
25–29	1373	6·5	1098	6·2	1135	6·4
>29	191	0·9	157	0·9	155	0·9
**Ethnicity**						
Hill Brahim	5014	23·6	4088	23·0	4296	24·1
Chhetri	3131	14·7	2605	14·6	2584	14·5
Janajaat	7427	34·9	6156	34·6	6136	34·5
Madhesi	1494	7·0	1323	7·4	1275	7·2
Dalit	3125	14·7	2680	15·1	2602	14·6
Muslim	684	3·2	614	3·5	580	3·3
Other	405	1·9	338	1·9	336	1·9
**Education at baseline**						
None	1680	7·9	1508	8·5	1438	8·1
Some primary	1192	5·6	1044	5·9	971	5·5
Completed primary	955	4·5	821	4·6	779	4·4
Some secondary	6076	28·6	5109	28·7	5099	28·6
Completed secondary	3675	17·3	3062	17·2	3101	17·4
More than secondary	7702	36·2	6260	35·2	6421	36·1
**Ever used any method of family planning at baseline**	7097	33·4	6166	34·6	6165	34·6
**Region at baseline**						
Hill or mountain	15883	74·6	13524	76·0	13358	75·0
Terai	5397	25·4	4280	24·0	4451	25·0
**Ever had abortion(s) before at baseline**	1042	4·9	876	4·9	907	5·1
**Follow-up characteristics**						
**Counseling timing**						
None	14771	69·4	10891	61·2	9323	52·4
Pre-discharge	6509	30·6	4058	22·8	2674	15·0
Post-discharge	··	··	1309	7·4	2917	16·4
Pre- and post-discharge	··	··	1546	8·7	2895	16·3
**Sex composition of living children at follow-up**	··	··				
No living children	··	··	136	0·8	102	0·6
Only daughters alive	··	··	5963	33·5	5892	33·1
A son alive	··	··	11705	65·7	11815	66·3
**Husband is away at follow-up**	··	··	4483	25·2	4467	25·1
**Index child died at follow-up**	··	··	273	1·5	311	1·8
**Months since delivery**	··	··				
<13	··	··	13604	76·4	8	0·0
13–18	··	··	4157	23·4	1786	10·0
19–24	··	··	42	0·2	1438	80·8
>24	··	··	1	0·0	1630	9·2
**Follow-up rate**	··	··	19626	92·2	19821	93·1

Although the percentage of women who never received counseling on postpartum family planning reduced over the two-year period, the rate of counseling was low. For example, women reporting no counseling declined from 69·4% at baseline, to 61·2% at year one, and then to 52·4% at year two. Only 30·6% of women reported counseling before discharge from the hospital. At year one, 16·1% of women had been counseled after discharge, including a mix of those not counseled at baseline (7·4%) and those counseled before discharge (8·7%). By year two, an additional 8·8% of women not counseled thus far received counseling, and 7·8% of women who had only received counseling before discharge were counseled post-discharge.

Table 2 presents the percentage of women with unmet need for modern contraception at year one and two follow-up surveys. Unmet need for modern contraception among postpartum women was high (54·2% at one year and 49.6% at two years post-delivery). Comparatively, we observed a higher rate of unmet need for spacing than for limiting. Unmet need was lower among those who received FP counseling at both pre-discharge and post-discharge (41·0% at year one and 42·8% at year two), highlighting potential positive impacts of counseling at both points in reducing unmet need.

**Table 2:** Number and percentage of women with unmet need for modern contraception.

	Year 1 (n = 17799)		Year 2 (n = 17809)	
	n	%	n	%
**Unmet need**				
Total	9654	54·2	8836	49·6
By counseling timing				
None	6239	57·3	4864	52·2
Pre-discharge	2098	51·7	1252	46·8
Post-discharge	683	52·2	1482	50·8
Pre- and post-discharge	634	41·0	1238	42·8
**Unmet need for spacing**				
Total	5434	30·5	5151	28·9
By counseling timing				
None	3346	30·7	2677	28·7
Pre-discharge	1298	32·0	771	28·8
Post-discharge	382	29·2	863	29·6
Pre- and post-discharge	408	26·4	840	29·0
**Unmet need for limiting**				
Total	4220	23·7	3685	20·7
By counseling timing				
None	2893	26·6	2187	23·5
Pre-discharge	800	19·7	481	18·0
Post-discharge	301	23·0	619	21·2
Pre- and post-discharge	226	14·6	398	13·8

Table 3 shows the percentage of women who received counseling at two years post-delivery by intervention arm. Approximately one-fourth fewer women were never counseled about FP in the baseline (control) period than during the intervention period. Unsurprisingly, more women who delivered during the intervention were counseled pre-discharge than women who delivered during the baseline period. However, 24·9% of women who delivered during the baseline period had been counseled post-discharge.

**Table 3:** Number and percentage of women who received counseling at year two postpartum, by intervention arm.

Counseling timing		Control (n = 6504)		Intervention (n = 11305)
	n	%	n	%
None	4454	68·5	4869	43·1
Pre-discharge	432	6·6	2242	19·8
Post-discharge	1322	20·3	1595	14·1
Pre- and post-discharge	296	4·6	2599	23·0

Table 4 presents the probability of a woman having unmet need for modern contraception in the postpartum period by counseling timing and sociodemographic characteristics. Women counseled in the pre-discharge period (adjusted odds ratio [aOR] 0·86; 95% CI: 0·80, 0·93) and in the post-discharge period (aOR 0·86; 95% CI: 0·79, 0·93) had the same likelihood of having unmet need in the postpartum period, suggesting that either timing will produce the same effect. However, women who received counseling in both the pre- and post-discharge period were 27% less likely than women who had not received counseling to have unmet need (95% CI: 0·67, 0·80), a 13 percentage point greater reduction than the other counseling groups.

**Table 4:** Odds ratios of unmet need for modern contraception by counseling timing and women’s sociodemographic characteristics, in the postpartum period (N = 21280 women; N = 35613 observations).

	OR	95% CI	p-value	aOR	95% CI	p-value
**Counseling timing (Ref: None)**						
Pre-discharge	0·85	0·80, 0·91	< .001	0·86	0·80, 0·93	< .001
Post-discharge	0·82	0·76, 0·88	< .001	0·86	0·79, 0·93	< .001
Pre- and post-discharge	0·68	0·63, 0·74	< .001	0·73	0·67, 0·80	< .001
**Husband is away at follow-up**	7·26	6·78, 7·76	< .001	7·58	7·07, 8·13	< .001
**Age at baseline (Ref: <21)**						
21–25	1·05	0·99, 1·12	.109	1·08	0·99, 1·19	.005
26–30	1·10	1·02, 1·18	.010	1·36	1·22, 1·52	< .001
31–35	1·12	1·01, 1·25	.036	1·61	1·40, 1·84	< .001
>35	1·03	0·81, 1·31	.817	1·68	1·37, 2·08	< .001
**Parity at baseline (Ref: 1)**						
2	1·01	0·96, 1·06	.760	1·04	0·97, 1·12	.185
3 or more	0·81	0·75, 0·88	< .001	0·83	0·74, 0·93	.003
**Ever used any method of family planning at baseline**	0·59	0·56, 0·62	< .001	0·55	0·52, 0·58	< .001
**Age at first marriage (Ref: <20)**						
20–24	1·00	0·95, 1·06	.862	0·87	0·81, 0·93	< .001
25–29	0·98	0·89, 1·09	.778	0·74	0·65, 0·85	< .001
>29	1·11	0·84, 1·47	.449	0·79	0·57, 1·08	.164
**Ethnicity (Ref: Hill Brahmin)**						
Chhetri	0·91	0·84, 0·99	.034	0·83	0·75, 0·91	< .001
Janajaat	0·77	0·72, 0·82	< .001	0·80	0·74, 0·87	< .001
Madhesi	0·93	0·83, 1·04	.190	1·12	0·99, 1·27	.073
Dalit	0·83	0·76, 0·90	< .001	0·84	0·76, 0·92	< .001
Muslim	0·99	0·85, 1·14	.856	1·04	0·88, 1·23	.622
Other	0·86	0·72, 1·04	.123	0·90	0·74, 1·09	.268
**Region at baseline (Ref: Hill or mountain)**						
Terai	0·97	0·86, 1·10	.682	0·93	0·82, 1·07	.286
**Ever had abortion(s) before at baseline**	0·88	0·79, 0·99	.032	1·04	0·91, 1·18	.606
**Education at baseline (Ref: None)**						
Some Primary	0·87	0·76, 0·99	.040	0·92	0·79, 1·06	.230
Completed primary	0·90	0·78, 1·04	.149	0·89	0·76, 1·04	.113
Some secondary	0·92	0·84, 1·02	.117	0·88	0·78, 0·98	.019
Completed secondary	0·98	0·88, 1·09	.661	0·94	0·83, 1·07	.311
More than secondary	1·13	1·02, 1·25	.017	1·14	1·01, 1·29	.041
**Sex composition of living children at follow-up (Ref: A son alive)**						
Only daughters alive	0·28	0·20, 0·38	< .001	0·26	0·17, 0·41	< .001
No living children	0·98	0·93, 1·03	.470	0·98	0·92, 1·04	.370
**Index child died at follow-up**	0·51	0·42, 0·61	< .001	0·88	0·68, 1·14	.311
**Months since delivery (Ref:** ≤**12)**						
13–18	0·92	0·86, 0·98	.010	0·89	0·83, 0·95	.003
19–24	0·81	0·78, 0·85	< .001	0·78	0·74, 0·82	< .001
>24	0·79	0·71, 0·87	< .001	0·76	0·68, 0·86	< .001

Notes: Sampling weights constructed using inverse probability weighting. All regression models adjusted for hospital and month fixed effects. Models include women’s random effect.

Abbreviations: OR = odds ratios; aOR = adjusted odds ratios.

Regarding sociodemographic characteristics, women whose husbands were away at follow-up were more than seven times more likely to have unmet need than women whose husbands were living at home (aOR 7·58; 95% CI: 7·07, 8·13). Women of older ages also had elevated odds of having unmet need, compared to women 20 years of age and younger. Compared to women in the Hill Brahmin caste, being in the Chhetri caste, Janajaat, or Dalit was associated with less than 0·85 times the odds of unmet need (aOR 0·83, 95% CI: 0·75, 0·91; aOR 0·80, 95% CI: 0·74, 0·87; aOR 0·84, 95% CI: 0·76, 0·92, respectively). Women who had more than a secondary education had an increased likelihood of having unmet need for modern methods compared to women with no education (aOR 1·14; 95% CI: 1·01, 1·29).

Women who had ever-used family planning were 45% less likely to have unmet need than women who had never used family planning (95% CI: 0·52, 0·58). Women with only daughters alive were 74% less likely to have unmet need than women with only a son alive (95% CI: 0·17, 0·41). Further, compared to women married before age 20, women married between the ages of 20 and 24 and between the ages of 25 and 29 were 13% (95% CI: 0·81, 0·93) and 26% (95% CI: 0·65, 0·85) less likely to have unmet need, respectively.

## Discussion

Women in our sample had high levels of unmet need for modern contraception in the postpartum period. Counseling either during pregnancy/before discharge or after discharge from the hospital was equally effective in reducing unmet need during the postpartum period. However, counseling women at both periods produced the greatest reduction in unmet need, suggesting that repeat exposure to messages and information about contraception during routing maternal services is the most effective strategy. Our study confirms the importance of providing FP counseling and information in maternal health services in Nepal, a country with high levels of unmet need, unintended pregnancy, and short-birth intervals.

Bivariate results from our study show that receipt of FP counseling either during antenatal and intrapartum care or postnatal care was low. Regarding pre-discharge counseling, this is surprising, given that the initiative focused on training providers to counsel women during routine antenatal care and before delivery, indicating a gap in the implementation of intervention. However, several studies in other settings have found that providers’ limited counseling on family planning is a major obstacle in integration of FP counseling into routine maternal health services [25]. For example, a large proportion of women in Ethiopia (78%) and Pakistan (87%) did not receive FP counseling during routine prenatal care, despite that the intervention’s focus was on integrating FP counseling into maternal care [26]. Providers, themselves, report that counseling pregnant women about contraception is difficult due to time constraints and limited staffing [27]. Nevertheless, our study supports the finding that providers routinely miss opportunities to offer FP information to women who have an unmet need and are at risk of unintended pregnancy.

We found support for the negative association between FP counseling before birth and subsequent unmet need for modern contraception. This is inconsistent with studies in other settings that found that FP counseling provided in the antenatal period does not influence contraceptive use in the 12 months after birth [15, 17, 28, 29]. However, our outcome measure, unmet need for modern contraception, is different from other studies, which tend to focus on use of any contraceptive method as the outcome. Unmet need for modern contraception is arguably as important, because it reflects the level of effective prevention of unintended pregnancy. Therefore, assessing how FP counseling meets women’s contraceptive needs, rather than the utilization of contraception alone, broadens our understanding about how to achieve universal public health goals while prioritizing women’s preferences. We also found support for the association between FP counseling before and after birth and subsequent lower unmet need for modern contraception. This finding is consistent with studies that found that providing birth spacing messages during antenatal and postnatal care significantly increases the use of postpartum contraception in the 10 to 12 months post-delivery and, therefore, reduce the unmet need [30].

Previous studies have documented several determinants of unmet need for contraception among postpartum women. However, most of these studies examined unmet need for any FP method rather than for modern methods [20, 31]. We identified determinants which are unique to the context of Nepal and the present study and considered some of those previously documented. For example, our study findings are consistent with two studies among married women of reproductive age that found that older women (age 30 or older) have higher odds of unmet need [20, 31]. However, contrary to other studies [20, 31], we did not find that Muslims had higher odds of unmet need for modern methods than hill-Brahmins. Instead, our study showed that Madhesi had the highest odds of unmet need compared to Brahmins. Unique to our study, we found that women whose husbands were away during the postpartum period had substantially higher odds (7·5) of unmet need. A common reason for nonuse of postpartum contraception is that a woman’s husband is traveling [32]. International migration, particularly to Gulf countries, has substantively increased in Nepal (from 2.3% in 2001 to 7.2% in 2011), but even more so among men [33]. Almost two million migrants overseas are men, and 32% of married women report that their husband work abroad [34]. The complexity of obtaining family planning services is heightened for women with migrant husbands, as the decision-making power is more likely to reside with mother-in-law as opposed to the women themselves [35]. As migration continues to rise, culturally appropriate counseling and/or interventions for this group of women who want to use contraception are essential to meeting their needs given the fragility of migration patterns and frequent and unexpected repatriations of overseas workers.

Women in our study sample were not nationally representative as they tended to have more years of schooling and younger than reproductive age women in Nepal [19]. We focused on women who lived within 24 hours travel distance of the hospital, and effects may be different for women in more remote areas. We also focused on large, tertiary level hospitals, excluding women who delivered outside of formal healthcare systems or at small healthcare centers. These women are likely to have an increased risk of unmet need [36]. Omission of information on the quality of counseling and its role in contraceptive use and unmet need is another limitation. Qualitative evidence from the PPIUD Nepal study suggests that the overall quality of the FP counselling during antenatal care was unsatisfactory, due to a lack of comprehensive information about FP, very crowded environments, limited time with the provider, and long wait times [37]. We also did not collect information on sexual activity that is critical for measuring unmet need in high migration for prolonged period in Nepal. Women only have unmet need if they are sexually active; however, all women in this sample were married–a typical proxy for sexual activity–and had a child in the last two years. In the absence of a measure of the frequency of women’s sexual activity, we relied on the standard DHS definition that considers women as having unmet need even though they are not currently (at a very specific point in time) sexually active [24].

## Conclusion

Family planning counseling before or after facility discharge reduced unmet need for modern methods during the 24-month postpartum period in Nepal. However, unmet need was much lower among women who received both pre- and post-discharge counseling, highlighting the importance of repeated FP counseling for effectively meeting the unmet need for modern methods during the postpartum period.

A major challenge for policy-makers and healthcare practitioners is how to support women in achieving healthy birth spacing and meeting their needs for postpartum contraception. The intervention increased counseling by 25 percentage points, but the overall level remained low. Heavy workload, group rather than individual counseling, and low priority accorded to counseling compared to other duties contributed to low coverage of counseling in Nepal. Interventions with dedicated counselors, together with counseling materials are likely to be more successful. Messages on contraception need to be an integral component of antenatal, intrapartum, and postpartum services in Nepal. The increase in institutional deliveries represents a prime opportunity to reach more pregnant and postpartum women [19]. However, there is an urgent need for interventions focused on training and supporting healthcare providers in FP counseling and testing strategies to improve the integration of FP and maternal health services.

## Supporting information

S1 Table Odds of being followed-up for the year one and year two surveys by selected background characteristics, among women who lived within 24 hours travel distance from the hospital at which they delivered and who were selected for follow-up. (DOCX)

S2 Table Predicted probabilities of being in the sample at each follow-up survey, total and by PPIUD insertion status at baseline, among women who lived within 24 hours travel distance from the hospital at which they delivered and who were selected for follow-up. (DOCX)

S1 Fig Distribution of sampling weights for the year one follow-up sample, among women who lived within 24 hours travel distance from the hospital at which they delivered. (DOCX)

S2 Fig Distribution of sampling weights for year two follow-up sample, among women who lived within 24 hours travel distance from the hospital at which they delivered. (DOCX)
