# Supplementary material for: Does family planning counseling reduce unmet need for modern contraception among postpartum women: Evidence from a stepped-wedge cluster randomized trial in Nepal
Source: PLoS One. 2021 Mar 26;16(3):e0249106. doi: 10.1371/journal.pone.0249106 (PMC7997001; doi:10.1371/journal.pone.0249106)
Supplement: S1 Table — (DOCX) [file pone.0249106.s001.docx]

**S1 Table. Odds of being followed-up for the year one and year two surveys by selected background characteristics, among women who lived within 24 hours travel distance from the hospital at which they delivered and who were selected for follow-up.**

|  | Year 1 follow-up sample | | Year 2 follow-up sample | |
| --- | --- | --- | --- | --- |
| Characteristics | OR | 95% CI | OR | 95% CI |
| **PPIUD Inserted** | 2·04*** | 1·70, 2·44 | 1·74*** | 1·46, 2·08 |
| **Age (Ref: Less than 21)** |  |  |  |  |
| 21-25 | 1·15*** | 1·05, 1·25 | 1·30*** | 1·20, 1·42 |
| 26-30 | 1·38*** | 1·23, 1·54 | 1·71*** | 1·53, 1·92 |
| 31-35 | 1·58*** | 1·33, 1·89 | 2·27*** | 1·87, 2·74 |
| 36 or older | 1·42* | 0·99, 2·02 | 2·31*** | 1·54, 3·46 |
| **Education (Ref: No schooling)** |  |  |  |  |
| Some primary | 0·93 | 0·66, 1·31 | 0·97 | 0·70, 1·34 |
| Completed primary | 0·94 | 0·66, 1·32 | 1·00 | 0·72, 1·39 |
| Some secondary | 1·10 | 0·80, 1·52 | 1·37* | 1·01, 1·85 |
| Completed Secondary | 1·15 | 0·83, 1·59 | 1·59*** | 1·16, 2·17 |
| More than secondary | 1·35* | 0·98, 1·87 | 1·89*** | 1·39, 2·58 |
| **Parity (Ref: 0-1)** |  |  |  |  |
| 2 | 1·12** | 1·03, 1·21 | 1·08* | 0·99, 1·18 |
| 3 or more | 1·04 | 0·90, 1·20 | 0·97 | 0·84, 1·12 |
| **Had abortion(s) before** | 0·94 | 0·80, 1·10 | 1·18* | 0·99, 1·41 |
| **Ethnicity (Ref: Hill Brahmin)** |  |  |  |  |
| Chhetri | 0·95 | 0·85, 1·06 | 0·82*** | 0·73, 0·91 |
| Janajaati | 0·99 | 0·90, 1·08 | 0·88*** | 0·80, 0·97 |
| Madhesi | 1·11 | 0·95, 1·29 | 0·97 | 0·83, 1·13 |
| Dalit | 0·87** | 0·78, 0·98 | 0·74*** | 0·66, 0·84 |
| Muslim | 1·28* | 0·99, 1·66 | 1·17 | 0·90, 1·51 |
| Other | 1·06 | 0·82, 1·37 | 0.91 | 0·70, 1·19 |
| **Married (Ref: Unmarried)** | 1·46 | 0·52, 4·13 | 1·09 | 0·35, 3·36 |
| **Male child born at index birth** | 1·06* | 0·99, 1·13 | 1·00 | 0·94, 1·07 |
| Constant | 1·94 | 0·66, 5·77 | 2·23 | 0·69, 7·16 |
| ****p* < 0.01; ***p* < 0.05; **p* < 0.1 | | | | |

Note: Original source for this table is Huber-Krum, S., Khadka, A., Rohr, J., Pradham, E., Puri, M., Maharjan, D., Joshi, S., Shah, I., & Canning, D. The effect of antenatal contraceptive counseling and IUD insertion services on modern contraceptive use and method mix in Nepal: Results from a stepped-wedge randomized controlled trial. In press at *Contraception*.
