# Supplementary material for: Does family planning counseling reduce unmet need for modern contraception among postpartum women: Evidence from a stepped-wedge cluster randomized trial in Nepal
Source: PLoS One. 2021 Mar 26;16(3):e0249106. doi: 10.1371/journal.pone.0249106 (PMC7997001; doi:10.1371/journal.pone.0249106)
Supplement: S2 Table — (DOCX) [file pone.0249106.s002.docx]

**S2 Table. Predicted probabilities of being in the sample at each follow-up survey, total and by PPIUD insertion status at baseline, among women who lived within 24 hours travel distance from the hospital at which they delivered and who were selected for follow-up.**

|  | Pr (follow-up) | Standard error | *p*-value | Lower limit | Upper limit |
| --- | --- | --- | --- | --- | --- |
| **Year 1 follow-up** | | | | | |
| Total | 0·810 | 0·003 | 0·000 | 0·805 | 0·815 |
| PPIUD not inserted | 0·805 | 0·003 | 0·000 | 0·800 | 0·810 |
| PPIUD inserted | 0·893 | 0·009 | 0·000 | 0·876 | 0·910 |
| **Year 2 follow-up** | | | | | |
| Total | 0·820 | 0·002 | 0·000 | 0·815 | 0·825 |
| PPIUD not inserted | 0·816 | 0·003 | 0·000 | 0·811 | 0·821 |
| PPIUD inserted | 0·885 | 0·009 | 0·000 | 0·867 | 0·902 |

Note: Original source for this table is Huber-Krum, S., Khadka, A., Rohr, J., Pradham, E., Puri, M., Maharjan, D., Joshi, S., Shah, I., & Canning, D. The effect of antenatal contraceptive counseling and IUD insertion services on modern contraceptive use and method mix in Nepal: Results from a stepped-wedge randomized controlled trial. In press at *Contraception*.
