# Supplementary material for: Does family planning counseling reduce unmet need for modern contraception among postpartum women: Evidence from a stepped-wedge cluster randomized trial in Nepal
Source: PLoS One. 2021 Mar 26;16(3):e0249106. doi: 10.1371/journal.pone.0249106 (PMC7997001; doi:10.1371/journal.pone.0249106)
Supplement: S2 Fig — (DOCX) [file pone.0249106.s004.docx]

**S2 Fig. Distribution of sampling weights for year two follow-up sample, among women who lived within 24 hours travel distance from the hospital at which they delivered.**

Note(s): Sampling weights were constructed by multiplying the predicted probability of year two follow-up for women who did not get the PPIUD inserted at baseline by 0·36 and for women who did get the PPIUD inserted at baseline by 1·00. Then, we took the inverse of the probabilities to generate the final weights.

Original source for this table is Huber-Krum, S., Khadka, A., Rohr, J., Pradham, E., Puri, M., Maharjan, D., Joshi, S., Shah, I., & Canning, D. The effect of antenatal contraceptive counseling and IUD insertion services on modern contraceptive use and method mix in Nepal: Results from a stepped-wedge randomized controlled trial. In press at *Contraception*.
